# Risk factors for cytomegalovirus infection in patients with antineutrophil cytoplasmic antibody-associated vasculitis

**DOI:** 10.1371/journal.pone.0218705

**Published:** 2019-07-10

**Authors:** Michiko Morishita, Ken-Ei Sada, Yoshinori Matsumoto, Keigo Hayashi, Yosuke Asano, Sumie Hiramatsu Asano, Keiji Ohashi, Yoshia Miyawaki, Eri Katsuyama, Haruki Watanabe, Tomoko Kawabata, Jun Wada

**Affiliations:** Department of Nephrology, Rheumatology, Endocrinology and Metabolism, Okayama University Graduate School of Medicine, Dentistry and Pharmaceutical Sciences, Okayama, Japan; University of Verona, ITALY

## Abstract

**Aims:**

Cytomegalovirus (CMV) infection under immunosuppression sometimes causes death. This study aimed to elucidate risk factors for CMV infection in patients with antineutrophil cytoplasmic antibody-associated vasculitis (AAV).

**Methods:**

Patients with AAV who underwent remission induction treatment at Okayama University Hospital between 2006 and 2016 were retrospectively analyzed. The primary outcome was the development of CMV infection within 3 months.

**Results:**

Of the 111 patients, 13 (11.7%) patients developed CMV infection. Patients with CMV infection were older (*p* = 0.030) and had a higher body mass index (*p* = 0.029) in comparison to those without CMV infection. A higher proportion had a severe form (*p* = 0.001) and granulomatosis with polyangiitis (GPA) (*p* = 0.001), as well as a higher Birmingham Vasculitis Activity Score (*p* = 0.018) and C-reactive protein (*p* = 0.018) levels at baseline. Using logistic regression analysis, severe form and GPA were independent risk factors (odds ratio [OR] = 9.68, 95% confidence interval [CI] = 1.92–60.23, and OR = 7.46, 95% CI = 1.46–47.60, respectively). In addition, patients with CMV infection were more likely than those without infection to be glucocorticoid-related diabetes mellitus (*p* = 0.025).

**Conclusion:**

Our study highlights disease severity and subgroups of AAV as risk factors for CMV infection.

## Introduction

Antineutrophil cytoplasmic antibody (ANCA)-associated vasculitis (AAV) is a systemic disorder associated with ANCA that predominantly affects small vessels and is classified into microscopic polyangiitis (MPA), granulomatosis with polyangiitis (GPA), and eosinophilic granulomatosis with polyangiitis (EGPA) [1]. Glucocorticoids (GCs) with immunosuppressants for remission induction have improved prognosis in patients with AAV, but bacterial, viral, and fungal infections are still a matter of concern, as they are a major cause of death [2, 3].

Cytomegalovirus (CMV) is a virus with a low pathogenicity that remains latent in the body of an infected host throughout its life [4, 5]. When the virus reactivates under immunosuppression, organs such as the lungs, gastrointestinal tract, and retina are involved, which sometimes causes death [4, 6, 7]. In transplant patients, CMV viremia should be treated to prevent the development of focal CMV disease and to improve the outcomes [8, 9]. Previous reports showed that white blood cell (WBC) counts, renal function, body mass index (BMI), hepatitis B surface antigen seropositivity, and age were risk factors for CMV infection in transplant recipients [5, 10–12].

The prevalence of CMV viremia and organ disease in patients with AAV have been reported as 0–5.8% [3, 13] and 0–3.5% [3, 13, 14], respectively. Although cyclophosphamide (CYC) use, GCs, renal dysfunction, older age, male sex, lymphopenia, pulmonary involvement, Birmingham Vasculitis Activity Score (BVAS), clinical grade category of rapidly progressive glomerulonephritis, and disease severity were associated with the development of all infections in patients with AAV [2, 3, 13–15], the risk factors for CMV infection have yet to be elucidated. The purpose of this study is to investigate the risk factors for CMV infection during remission induction treatment in patients with AAV.

## Patients and methods

### Patient selection

We retrospectively reviewed the medical records of consecutive inpatients with AAV from 2006 to 2016 at Okayama University Hospital. Enrolled patients were fulfilled the criteria for primary systemic vasculitis as proposed by the European Medicines Agency (EMA) algorithm [16], hospitalized for remission induction treatment for AAV, and had the CMV antigenemia test performed at least once within the following 3 months.

### Data collection

The data of enrolled patients at the initiation of remission induction therapy included their demographic information, comorbidities, disease classification and severity, BVAS [17], laboratory data, ANCA specificity, and treatment status. Treatment status included the initial dosage of GCs and concomitant use of methylprednisolone pulse and immunosuppressants. Patients were also evaluated within 3 months for the following parameters: disease activity, infectious complications, and GC-related adverse events. The disease severity was classified as localized, early systemic, generalized, or severe based on the European League Against Rheumatism recommendation for conducting a clinical study in systemic vasculitis [18]. According to a previous study, organ failure (the presence of any of the following BVAS manifestations: massive hemoptysis/alveolar hemorrhage, respiratory failure, congestive cardiac failure, ischemic abdominal pain, or cerebrovascular accident) was defined as severe. Threatened vital organ function (the presence of any of the following BVAS manifestations: sudden visual loss, blurred vision, retinal changes, conductive deafness, sensorineural hearing loss, ischemic cardiac pain, cardiomyopathy, peritonitis, bloody diarrhea, meningitis, organic confusion, seizures, cord lesions, cranial nerve palsy, sensory peripheral neuropathy, or motor mononeuritis multiplex) was defined as generalized [19]. In GPA, cases with only ear, nose, and throat (ENT) and/or chest involvement were classified as localized. Other cases were classified as early systemic. In cases with renal involvement, serum creatinine levels were also used to classify disease severity as localized and early systemic (<125 μmol/l [1.41 mg/dL]), generalized (<500 μmol/L [5.66 mg/dL]), and severe (≥500 μmol/L [5.66 mg/dL]) [19]. Remission was defined as the complete absence of disease activity attributable to active vasculitis. The absence of disease activity was determined systematically using a BVAS of 0. As for GC-related adverse events, GC-related diabetes mellitus (GCDM) was defined as the initiation of a hypoglycemic agent after the initiation of GC regardless of a complication with DM at baseline.

This study was conducted according to the Declaration of Helsinki and the ethical guidelines for epidemiologic research in Japan. The study protocol was approved by the ethics committees of Okayama University Graduate School of Medicine, Dentistry and Pharmaceutical Sciences (authorization number: Ken 1711–016). All patients provided informed consent to participate in the study as well as permission to have their data published.

### Treatment for AAV and management of CMV infection

For remission induction of AAV, the patients were treated according to the European League Against Rheumatism recommendation [20]. Generally, they were treated with GC and pulse CYC based on CYCLOPS protocol [21], while the treatments were determined at the discretion of the attending physicians finally.

After initiation of the remission induction treatment, we performed the CMV antigenemia test every two weeks. When CMV antigenemia test showed >0 and <5 CMV pp65 antigen-positive cells/50,000 WBCs, CMV antigenemia test was performed weekly. If CMV antigenemia test showed ≥5 CMV pp65 antigen-positive cells/50,000 WBCs, we started treatment as CMV infection.

### Outcomes

The primary outcome of this study was the development of CMV infection within 3 months after initiation of treatment for AAV. CMV infection was defined as viremia (≥5 CMV pp65 antigen-positive cells/50,000 WBCs), CMV syndrome (at least two of the following: fever, malaise, leucopenia or neutropenia, atypical lymphocyte, thrombocytopenia, elevation of hepatic aminotransferases), or focal CMV disease, such as retinitis, pneumonitis, hepatitis, or gastrointestinal disease, based on previous reports [10, 22–24].

### Statistical analyses

The descriptive statistics are expressed as the median and interquartile range (IQR) unless otherwise specified. Continuous variables were compared using Student’s t-test or the Mann-Whitney U test depending on data distribution, and categorical variables were compared outcomes between patients with and without CMV infection using Fisher’s direct probability test. In multivariate logistic regression analysis, candidate risk factors were selected according to the results of univariate analysis and the findings of previous reports [2, 3, 5, 10, 12, 15]. We also performed sensitivity analysis changing some candidate risk factors. The tests were two-tailed, and differences of *p* < 0.05 were considered significant. All statistical analyses were performed using the Statistical Package of JMP for Windows software program, version 11.2.0 (SAS Institute Inc., Cary, NC, USA).

## Results

### Patient characteristics

Of 162 inpatients with AAV, 130 patients were hospitalized for remission induction therapy but 19 were excluded because of a lack of the CMV antigenemia test within 3 months after initiation of the treatment. Of those 111 patients with the CMV antigenemia test, 104 patients (93.7%) were performed within 1 month after initiation of the treatment. The baseline characteristics of 111 patients are summarized in Table 1. The median age of the enrolled patients was 70 (IQR, 63–77) years, and 67.6% were women. The median BMI was 21.2 (IQR, 19.2–23.5) kg/m^2^ and 18.0% of patients were complicated with diabetes at the initiation of the treatment. Using the EMA algorithm, 64 were classified as MPA, 22 as GPA, 12 as EGPA, and 13 as unclassifiable AAV. Myeloperoxidase-ANCA and proteinase 3-ANCA were positive in 81.1% and 8.1% of patients, respectively. The median BVAS was 14 (IQR, 11–18), and interstitial lung disease was found in 41.4% of patients. The median initial daily prednisolone dose was 40 (IQR, 30–50) mg. Concomitant immunosuppressants were used in 92 (82.9%) patients (CYC in 79, azathioprine in 10, and rituximab in 3). Only one patient did not achieve remission and died with aspiration pneumonia during the observational period. GCDM developed in 53 (47.7%) patients during the observational period.

**Table 1:** The baseline characteristics and treatments of 111 patients.

Men / women, n	36 / 75
Age, years	70 [63–77]
BMI, kg/m^2^	21.2 [19.2–23.5]
ILD, n (%)	46 (41.4)
Classification	
MPA, n (%)	64 (57.7)
GPA, n (%)	22 (19.8)
EGPA, n (%)	12 (10.8)
Unclassifiable, n (%)	13 (11.7)
Severity	
Localized, n (%)	1 (0.9)
Early systemic, n (%)	32 (28.8)
Generalized, n (%)	56 (50.5)
Severe, n (%)	22 (19.8)
Disease newly diagnosed at enrollment, n (%)	100 (90.0)
Complication of diabetes, n (%)	20 (18.0)
MPO-ANCA positive, n (%)	90 (81.1)
PR3-ANCA positive, n (%)	9 (8.1)
BVAS	14 [11–18]
Treatment	
mPSL pulse use, n (%)	35 (31.5)
Initial dose of PSL, mg/day	40 [30–50]
PSL/Weight, mg/kg	0.832 [0.669–0.957]
New concomitant use of immunosuppressant, n	92
Initiation of cyclophosphamide, n	79

Data are presented median and numbers in brackets indicate interquartile range (IQR). BMI, body mass index; ILD, interstitial lung disease; MPA, microscopic polyangiitis; GPA, granulomatosis with polyangiitis; EGPA, eosinophilic granulomatosis with polyangiitis; BVAS, Birmingham Vasculitis Activity Score; ANCA, antineutrophil cytoplasmic antibody; mPSL, methylprednisolone.

### Risk factors for CMV infection

The mean number of the CMV antigenemia measurements was 1.9 by 1 month and 4.4 by 3 months in 111 patients. During the observation period, 13 of 111 patients (11.7%) was diagnosed as a CMV infection at a median of 18 (IQR, 12.5–34) days after the initiation of the AAV treatment. Median CMV pp65 antigen-positive cells/50,000 WBCs was 7.0 (IQR, 5.5–9.5). Median WBC count was 8620/μL (IQR, 7370–11425) and median lymphocyte count was 900/μL (IQR, 330–1900) at the development of CMV infection. All 13 patients had CMV viremia, and no patient had CMV syndrome and focal CMV disease. All patients were treated with ganciclovir, and viremia disappeared in 8 (61%) patients one week after and 10 (77%) patients two weeks after the initiation of the treatment.

The baseline characteristics of patients with and without CMV infection for the univariate analysis are summarized in Table 2. Patients with CMV infection were significantly older (76 vs. 69 years, *p* = 0.030), had a higher BMI (23.0 vs. 20.7 kg/m^2^, *p* = 0.029), and included a higher proportion of severe form (53.8 vs. 15.3%, *p* = 0.001) than those without CMV infection. Moreover, the proportion of GPA was higher in patients with CMV infection than in those without CMV infection (53.8 vs. 15.3%, *p* = 0.001), while ANCA specificity was comparable. BVAS and C-reactive protein (CRP) levels were higher in patients with CMV infection than those without (16 vs. 13, *p* = 0.018 and 11.2 vs. 5.3 mg/dL, *p* = 0.018, respectively). For the multivariate analysis, the following factors were selected based on our univariate analysis and previous reports; age, BMI, GPA, severe form (vs. other disease severities), hemoglobin A1c (HbA1c), and CRP (Table 3). In a logistic regression analysis, GPA and severe form were identified as significant risk factors for CMV infection (odds ratio [OR] = 7.46, 95% confidence interval [CI] = 1.46–47.60, and OR = 9.68, 95% CI = 1.92–60.23, respectively). In patients categorized as severe form, chest symptom was more frequent (81.9% vs 20.2%, *p* < 0.0001) and eGFR was lower (16.1 vs 50.6 mL/min/1.73m^2^) than in the other form patients significantly. Because disease severity had a strong correlation with eGFR and BVAS, we performed sensitivity analysis using two models. In the first model, age, BMI, GPA, eGFR, HbA1c, and CRP were evaluated and the only GPA was identified as a significant risk factor for CMV infection (*p* = 0.012). In the second model using age, BMI, GPA, BVAS, HbA1c, and CRP, it was also confirmed that GPA was the only risk factors for CMV infection.

**Table 2:** Comparison of the baseline characteristics and treatments of the patients with and without cytomegalovirus (CMV) infection.

	With CMV infection (n = 13)	Without CMV infection (n = 98)	*p* value
Men / women, n	6 / 7	30 / 68	0.260
Age, years	76 [72–80]	69 [63–76]	0.030
BMI, kg/m^2^	23.0 [21.4–25.1]	20.7 [18.9–23.4]	0.029
HBs Ag positive, n (%)	0 (0)	1 (1.0)	0.720
ILD, n (%)	4 (30.1)	42 (42.9)	0.406
Classification			
MPA, n (%)	4 (30.8)	60 (61.2)	0.037
GPA, n (%)	7 (53.8)	15 (15.3)	0.001
EGPA, n (%)	2 (15.4)	10 (10.2)	0.572
Unclassifiable, n (%)	0 (0)	13 (13.3)	0.162
Severity			
Localized, n (%)	0 (0)	1 (0.9)	0.715
Early systemic, n (%)	2 (1.8)	30 (27.0)	0.255
Generalized, n (%)	4 (30.8)	52 (53.1)	0.131
Severe, n (%)	7 (53.8)	15 (15.3)	0.001
Disease newly diagnosed at enrollment, n (%)	1 (7.7)	10 (10.2)	0.776
Complication of diabetes, n (%)	3 (23.1)	17 (17.3)	0.630
MPO-ANCA positive, n (%)	11 (84.6)	79 (80.6)	0.379
PR3-ANCA positive, n (%)	0 (0)	9 (9.2)	0.246
BVAS	16 [14–23]	13 [9–18]	0.018
Laboratory data			
WBC, /μL	13000 [10035–18390]	10200 [7980–13310]	0.097
Neu, /μL	9337.5 [7719.5–12260]	7791.5 [5845–10450]	0.174
Lym, /μL	1158.5 [900.5–1822.75]	1241 [974–1676.5]	0.782
HbA1c, %	6.4 [6.0–6.7]	6.0 [5.8–6.5]	0.197
Cre, mg/dL	2.01 [0.96–3.02]	1.03 [0.68–2.26]	0.076
eGFR, mL/min/1.73m^2^	26.38 [13.07–56.43]	46.04 [18.42–71.56]	0.105
CRP, mg/dL	11.2 [5.36–17.02]	5.32 [1.56–11.45]	0.018
Treatment			
mPSL pulse use, n (%)	6 (46.2)	29 (29.6)	0.227
Initial dose of PSL, mg/day	40 [37.5–50]	40 [30–50]	0.786
PSL/Weight, mg/kg	0.798 [0.584–0.925]	0.840 [0.672–0.965]	0.329
New concomitant use of immunosuppressant, n	10	82	0.544
Initiation of cyclophosphamide, n	10	69	0.626

Data are presented median and numbers in brackets indicate interquartile range (IQR). CMV, cytomegalovirus; BMI, body mass index; HBs Ag, hepatitis B surface antigen; ILD, interstitial lung disease; MPA, microscopic polyangiitis; GPA, granulomatosis with polyangiitis; EGPA, eosinophilic granulomatosis with polyangiitis; ANCA, antineutrophil cytoplasmic antibody; BVAS, Birmingham Vasculitis Activity Score; WBC, white blood cells; Neu, neutrophils; Lym, lymphocytes; HbA1c, hemoglobin A1c; Cre, creatinine; eGFR, estimated glomerular filtration rate; CRP, C-reactive protein; mPSL, methylprednisolone.

**Table 3:** Risk factors for development of CMV infection within 3 months after the initiation of the therapy.

	Odds ratio (95% CI)	*p* value
Age, years	1.05 (0.96–1.16)	0.271
BMI, kg/m^2^	1.19 (0.94–1.53)	0.142
GPA	7.46 (1.46–47.60)	0.015
Severe form (vs. other disease severities)	9.68 (1.92–60.23)	0.006
HbA1c, %	0.90 (0.20–3.38)	0.883
CRP, mg/dL	1.08 (0.95–1.23)	0.249

Odds ratios, 95% CI and *p* values were calculated using the logistic regression analysis. CMV, cytomegalovirus; BMI, body mass index; GPA, granulomatosis with polyangiitis; HbA1c, hemoglobin A1c; CRP, C-reactive protein.

The patients with CMV infection developed GCDM more frequently than patients without CMV infection (76.9% vs. 43.9%, *p* = 0.025). These differences were larger among patients without a complication of DM at baseline (9 of 10 [90.0%] vs. 34 of 81 [42.0%], *p* = 0.004).

## Discussion

This is the first report of the risk factors for CMV infection in patients with AAV. Overall, 11.7% (13 of 111) of patients with AAV developed CMV infection.

CMV infection occurred more frequently in the present study than in the previous two studies on AAV [13, 14]. These reports for patients with GPA included much younger patients than those included in our study (50.2 years and 51 years in the previous studies, respectively, and 72.2 years in the present study), and they focused on focal CMV diseases. The high frequency of CMV infection in the present study might be related to the definition of CMV infection that included CMV viremia. Actually, the rate of CMV infections in other previous reports (8.3%) [3] was comparable to that of our report because CMV viremia was included as CMV infection. In the transplantation field, CMV viremia is treated as CMV infection to prevent focal CMV disease and to improve the outcomes [8, 9]. All 13 patients had CMV viremia, and no patient had focal CMV disease. Because no deaths related to CMV infection were found in the present study, monitoring and treatment for CMV viremia might be important to improve the prognosis of patients with AAV.

GPA and severe form emerged as independent factors by multivariate analysis. Previous reports showed that a severe form of AAV was an independent risk factor for severe infections [3]. We confirmed that a severe form of AAV was also an independent risk factor for isolated CMV infection alone. In the absence of prior reports, GPA may be a specific risk factor for CMV infection. In our study, the GPA patients had ENT symptoms more frequent than the other patients (63.6% vs 7.9%, *p* < 0.0001). CMV sinusitis and nasal polyposis have been reported in patients with HIV and other immunosuppressed states [25–29]. Therefore, the presence of upper respiratory inflammation in GPA patients on immunosuppressive treatment might be related to the development of CMV infection. In the present study, patients with CMV infection developed GCDM more frequently, but there was no significant difference in HbA1c and DM at baseline. Although a previous report showed that diabetes was related to CMV infection in patients after renal transplantation [30], development of the infection might be influenced more strongly by glucose tolerance to steroids than by the presence of DM at baseline.

Several limitations of the present study warrant mention. First, we might have underestimated the frequency of CMV infection because the CMV antigenemia test was not performed systematically. However, the CMV antigenemia test was performed in most patients within 1 month, and CMV viremia was diagnosed within 1 month; therefore, underestimation might not be critical. Second, our patients were treated according to the treatment protocol, and treatment was relatively uniform; therefore, treatment-related factors, such as GC dosage, could not be sufficiently evaluated in the present study. However, this is a strength of our study because we were able to determine which patients required careful monitoring for CMV before treatment. Finally, evaluation of anti-CMV IgG before treatment was not performed in the present study. However, a previous report showed that about 95% of the elderly population were anti-CMV IgG-positive [31]. Because AAV frequently develops in elderly patients, monitoring for CMV viremia should be performed without the evaluation of anti-CMV IgG.

In conclusion, our study highlights severe form of AAV and GPA as independent risk factors for CMV infection in patients with AAV.
